# Human Polyclonal Antibodies Produced from Transchromosomal Bovine Provides Prophylactic and Therapeutic Protections Against Zika Virus Infection in *STAT2* KO Syrian Hamsters

**DOI:** 10.3390/v11020092

**Published:** 2019-01-22

**Authors:** Venkatraman Siddharthan, Jinxin Miao, Arnaud J Van Wettere, Rong Li, Hua Wu, Eddie Sullivan, Jinan Jiao, Jay W. Hooper, David Safronetz, John D. Morrey, Justin G. Julander, Zhongde Wang

**Affiliations:** 1 Department of Animal, Dairy, and Veterinary Sciences, Utah State University, 5600 Old Main Hill, Logan, UT 84322, USA; jinxin.miao@yahoo.com (J.M.); lirong14@gmail.com (R.L.); john.morrey@usu.edu (J.D.M.); justin.julander@usu.edu (J.G.J.); 2 Institute for Antiviral Research, and Utah State University, 5600 Old Main Hill, Logan, UT 84322, USA; 3 Department of Pathology, School of Basic Medical Sciences, Zhengzhou University, Zhengzhou 450066, China; 4 Utah Veterinary Diagnostics Laboratory, Department of Animal, Dairy, and Veterinary Sciences, Utah State University, 5600 Old Main Hill, Logan, UT 84322, USA; arnaud.vanwettere@aggiemail.usu.edu; 5 SAB Biotherapeutics, Sioux Falls, SD 57104, USA; hwu@sabbiotherapeutics.com (H.W.); esullivan@sabbiotherapeutics.com (E.S.); jjiao@sabbiotherapeutics.com (J.J.); 6 Virology Division, United States Army Medical Research Institute of Infectious Diseases, Fort Detrick, MD 21702, USA; jay.w.hooper.civ@mail.mil; 7 Zoonotic Diseases and Special Pathogens, National Microbiology Laboratory, Public Health Agency of Canada, Winnipeg, MB R3E, Canada; david.safronetz@canada.ca

**Keywords:** *STAT2* KO hamster, transchromosomal bovine antibody, Zika virus, therapeutic, testis

## Abstract

Zika virus (ZIKV) infection can cause severe congenital diseases, such as microcephaly, ocular defects and arthrogryposis in fetuses, and Guillain–Barré syndrome in adults. Efficacious therapeutic treatments for infected patients, as well as prophylactic treatments to prevent new infections are needed for combating ZIKV infection. Here, we report that ZIKV-specific human polyclonal antibodies (SAB-155), elicited in transchromosomal bovine (TcB), provide significant protection from infection by ZIKV in *STAT2* knockout (KO) golden Syrian hamsters both prophylactically and therapeutically. These antibodies also prevent testicular lesions in this hamster model. Our data indicate that antibody-mediated immunotherapy is effective in treating ZIKV infection. Because suitable quantities of highly potent human polyclonal antibodies can be quickly produced from the TcB system against ZIKV and have demonstrated therapeutic efficacy in a small animal model, they have the potential as an effective countermeasure against ZIKV infection.

## 1. Introduction

Zika virus (ZIKV) is a mosquito-borne flavivirus in the family Flaviviridae, which not only affects human adults by causing Guillain–Barré syndrome but also induces microcephaly and death in congenitally exposed fetuses [1,2]. The recent ZIKV outbreak in South America has resulted in an unprecedented large number of infection cases [3]. ZIKV has emerged as a public health threat. Currently, there are no countermeasures available for the prevention or treatment of ZIKV infection, except for symptom relief management. Vaccine development is still at its early stages and it is unknown when an approved ZIKV vaccine will be available. 

To address the unmet biomedical needs for the production of therapeutic antibodies, SAB Biotherapeutics, Inc. has developed a transchromosomic (Tc) bovine platform with the capability to produce large quantities of fully-human polyclonal antibodies [4]. In this Tc bovine (TcB) system, bovine immunoglobulin genes were genetically inactivated and the Ig functions were reconstituted by a human artificial chromosome (HAC) comprising the entire unrearranged human immunoglobulin repertoire. It has been demonstrated that not only physiological levels of fully-human polyclonal antibodies can be produced in the blood of TcB but also that TcB can be hyperimmunized with an antigen of choice to produce highly potent antigen-specific human polyclonal antibodies. Some of these antibodies have been successfully used to treat a list of viral and bacterial infections [5].

We recently demonstrated that anti-ZIKV human polyclonal antibodies (SAB-155) produced from TcB administered at −1 and +1 dpi provided 100% protection against ZIKV infection in wild type mice treated with an anti-interferon receptor antibody and in homozygous *Ifnar1* knockout (*Ifnar^-/-^*) C57BL/6 mice, and eliminated ZIKV induced tissue damages in the brain and testis [6]. In the meantime, as an effort to develop novel animal models of viral infection, we have developed a signal transducer and activator of transcription 2 (*STAT2*) gene knockout (KO) golden Syrian hamster [7]. Compared to the commonly used A129, *Ifnar^-/-^*, or the AG129 mice as models of viral infections, in which either the type I interferon (A129 and *Ifnar^-/-^* mice) or both type I and type II interferon receptors are knocked out (AG129 mice), *STAT2* KO hamsters are only partially defective in type I interferon signaling, thus they are less immunocompromised than the above-mentioned mouse models. Furthermore, because ZIKV, as well as several other flaviviruses, exerts its infectivity in humans [8,9] through targeting human STAT2 protein to inactivate human type I interferon responses, infection of *STAT2* KO hamsters by ZIKV would mimic the innate immune responses in humans upon ZIKV infection. Indeed, we recently have demonstrated that *STAT2* KO hamsters are highly susceptible to ZIKV infection. By using this novel *STAT2* KO hamster, the first non-murine rodent model of viral infection, we recently demonstrated that infection of pregnant hamsters leads to the vertical transmission of ZIKV to the uterus, placenta, and immune privileged sites, such as the testes and fetal brain [10].

In the present study, we evaluated the anti-ZIKV human polyclonal antibodies (SAB-155) produced from TcB both as therapeutic and prophylactic treatments for ZIKV infection in *STAT2* KO hamsters. We demonstrated that both treatments with SAB-155 provide significant protection from lethal infection by ZIKV in the *STAT2* KO hamster model. SAB-155 also protected the testes from ZIKV infection when the animals were treated as late as three days post-infection (dpi).

## 2. Materials and Methods

### 2.1. Virus

The ZIKV used in this study was the PRVABC59 ZIKV strain that was originally isolated in Puerto Rico from the blood of a human patient in December 2015. The virus was provided by Barbara Johnson (Center for Disease Control and Prevention, Fort Collins, USA). A virus stock was prepared by two passages in Vero cells and had a titer of 10^7.5^ 50% cell culture infectious doses (CCID_50_)/mL [10]. 

### 2.2. Production of Anti-ZIKV Human Polyclonal Antibodies SAB-155 from Transchromosomal Bovine

The TcB used in this study carries a human artificial chromosome (HAC) comprising the entire human Ig gene repertoire in the germline genomic configuration in the genetic background that the endogenous bovine immunoglobulin genes, *IGHM; IGHML1; IGL,* were sequentially knocked out (*IGHM^–/–^; IGHML1^–/–^; IGL^–/–^)* [4]. The generation of anti-ZIKV human polyclonal antibodies SAB-155 from TcB was described previously [6,11]. Briefly, a plasmid DNA (pDNA) encoding a full-length ZIKV prME gene described by Hooper et al. [6] was used as a vaccine to immunize TcB. The TcB was hyperimmunized 4 times (V1–V4) at 3-week intervals with the antigen at 12 mg per animal per vaccination by using a PharmaJet Stratis^®^ IM injection device as previously described [6]. ZIKV-specific antibodies, termed SAB-155, were purified from the plasma collected from hyperimmunized animals. Negative control antibodies used in this study were human polyclonal antibodies purified from the sera of the same TcB before ZIKV immunization.

### 2.3. STAT2 KO Golden Syrian Hamsters

*STAT2* KO golden Syrian hamsters produced in-house [7] were used in this study at 5 to 6 weeks of age. For infection, ~70 pfu of ZIKV was administered to *STAT2* KO hamsters by the subcutaneous (s.c.) route in the inguinal area. For prophylactic and therapeutic treatments, different doses of SAB-155, in the range of 1 mg to 400 mg/kg were administered via intraperitoneal (i.p.) injections. 

### 2.4. Serum and Tissue Collection

Serum was separated from whole blood using serum separator tubes by centrifugation at 12.5 × 10^3^ g for 5 min and stored −80 °C before use. Testicular tissues were collected for quantifying ZIKV RNA, ZIKV-immunohistochemistry (IHC) assays, and histological analysis. For RT-PCR, tissue was homogenized in Trizol™ in a microcentrifuge with a Teflon pestle, and frozen until RNA extraction [10,12]. Either the other testis from the same animal or remaining tissue from the same testis was fixed using freshly prepared 4% paraformaldehyde. Prior publications from our laboratories and others reiterated that the testis was one of the target organs for ZIKV infection in rodents [13,14,15] therefore we used the testis as one of the major organs to evaluate with the TcB antibody. Both sexes were susceptible for disease leading to a moribund state. Twenty-four hours later the tissues were rinsed once in PBS and placed in 70% ethanol. Paraffin-embedded tissues were sectioned at 5 μM thickness and processed for hematoxylin-eosin (HE) staining and ZIKV-immunoreactive (ZIKV-ir) assays. 

### 2.5. ZIKV Plaque Reduction Neutralization Test (PRNT)

The neutralizing activities of hamster serum antibodies were determined by a plaque reduction assay [16]. After incubation at 56 °C for 30 min, 2-fold serial dilutions of serum were mixed and incubated at 4 °C overnight with a stock of ZIKV (100 PFU), with a final starting dilution of 1:10 of each serum sample. A total volume of 200 µl serum with PBS and ZIKV mixture was added to each well of a twelve-well plate containing Vero 76 cells at 90% confluence. The plate was incubated for 1 h with periodic rocking at 37 °C. One ml of agarose overlay media was added and incubated at 37 °C. Three days later, the cells were stained with a crystal violet solution containing formaldehyde and the plaques were counted. The inverse of the dilution that caused a 50% reduction was reported as the 50% plaque reduction neutralization titer (PRNT_50_). The titer of the infectious virus was similarly determined, except that no serum was added to the sample. 

### 2.6. ZIKV RT-PCR

Serum RNA was extracted using QIAamp Viral RNA mini-kit from Qiagen (Qiagen, Germantown, MD, USA). Fifty to 100 mg of freshly isolated tissue or 0.1 mL of tissue homogenized with a pestle was added to 900 µL of Trizol Reagent™ (Molecular Research Center, Cincinnati, OH, USA) for RNA extraction. RNA was suspended in RNAsecure (Life Technologies, Carlsbad, CA, USA) and was amplified by a quantitative RT-PCR. Ten microliters of 2 Å~ SensiFAST^TM^ Probe No-ROX One-step Mix, (Bioline, Memphis, TN, USA) with polymerase and reverse transcriptase, along with Malaysian ZIKV primers (20 μM) (5′-CTGTGGCATGAACCCAATAG-3′, 3′-ATCCCATAGAGCACCACTCC-5′) and probe (20 μM) (5′-FAMCCACGCTCCAGCTGCAAAGG-3′TAMRA) labeled with a FAM fluorophore and TAMRA quencher (GenScript, Piscataway, NJ, USA), respectively, were mixed with 2 μL of extracted RNA. Each sample was run in duplicate and relative genome equivalents were averaged. GAPDH primers (20 μm) and probe (20 μm) were also included. ZIKV RNA was reverse transcribed for 10 min at 45 °C, then heated to 95 °C for 2 min. The PCR amplification cycles were 40 cycles at 95 °C for 5 s, and 60 °C for 20 s. Standard curves of ZIKV RNA and GAPDH RNA were generated with serial dilutions of synthetic RNA (GenScript, Piscataway, NJ, USA) of the target sequence (accession HQ234499.1) diluted in normal mouse total RNA (Agilent Technologies, Santa Clara, CA, USA). The thermocycler was Mic-2 qPCR (Bio Molecular Systems, Taunton, MA, USA). The relative number of ZIKV RNA was determined from the standard curve and was normalized with relative total RNA calculated from the GAPDH standard curve. We had previously validated that GAPDH levels were not modified by ZIKV infection. The limit of detection was calculated based on the titer values from sham or uninfected testis tissue. 

### 2.7. Immunohistochemistry (IHC) and Histology

Following de-paraffinization, tissue sections were blocked with normal goat serum for 60 min at room temperature [17]. Either mouse anti-flavivirus group antigen monoclonal antibody (Millipore, Temecula, CA, USA) or mouse monoclonal antibody to ZIKV NS1 protein (Aalto Bio Reagents, Dublin, Ireland) was incubated on the tissues for overnight. After a brief wash with PBS, sections were incubated with a fluorescence-conjugated secondary antibody (Invitrogen, Grand Island, NY, USA) at room temperature for 2 h. Images were captured using Zeiss microscope, AxioVision 4.0.1 and processed using Adobe Photoshop. Hematoxylin and eosin (H&E) staining was performed to study the morphological diagnoses (Table 1) and it was read by a veterinary anatomic pathologist (Dr. A.V.W).

### 2.8. Ethics Statement

The experiments were conducted in strict accordance with guidelines of the AAALAC accredited Laboratory Animal Research Center at Utah State University and approved by the Institutional Animal Care and Use Committee of Utah State University (IACUC Protocol: 2667; approval date: 27 September 2016). 

## 3. Results

### 3.1. ZIKV Infection in STAT2 KO Hamsters

**Figure 1:**
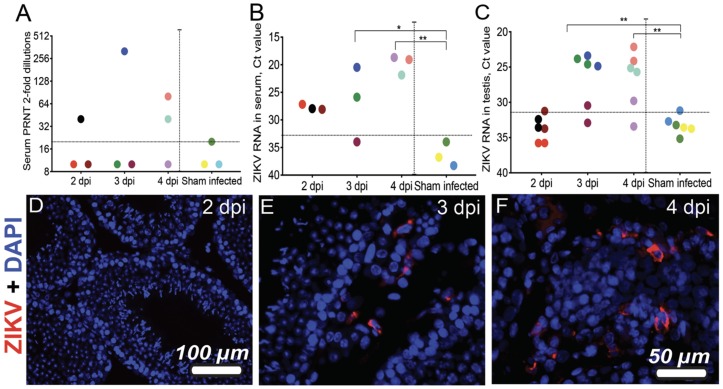
Defining Zika virus (ZIKV) infection on *STAT2* KO hamsters during acute infection. Serum PRNT (**A**), ZIKV RNA titers in serum (**B**) and testes (Ct values) (**C**), and immunohistochemistry of ZIKV antigens in testes at 2, 3, and 4 dpi (**D**–**F**). Three animals were processed for each time point (**A**–**C**). Tissues from both right and left testes were processed for RT-PCR (C) and representative IHC images are shown with ZIKV infection (**D**–**F**). **, *p* < 0.005 by one-way ANOVA, sham infection compared with ZIKV infection. Horizonal dashed lines designate limit of detection in Figure 1A–C based on sham infection; vertical dashed lines separate infected animals from sham infected ones. Each of the animals were color-coded in **A**–**C**.

To characterize the activity of neutralizing antibodies, the kinetics of viremia and viral presence in the testis of *STAT2 KO* hamsters following ZIKV infection, we infected three age-matched (6 weeks of age) male hamsters via the s.c route with PRVABC69 (~70 pfu) ZIKV. Sera were collected at 2, 3, and 4.5 dpi for detecting neutralizing antibody titers. Based on the PRNT_50_ data (Figure 1A), sera assayed at these time-points had detectable levels of ZIKV-specific neutralizing antibody, indicating that infection can elicit a neutralizing antibody response in *STAT2* KO hamsters. Significant serum viremia was also found at 3 dpi (2/3 animals) and 4.5 dpi (3/3) animals (Figure 1B). To investigate ZIKV infection in testis, testicles were harvested at 2, 3, and 4.5 dpi for ZIKV RNA detection and immunohistochemistry analysis. ZIKV RNA levels were significantly higher in two animals on both the right and left testis at 3 and 4.5 dpi than at 2 dpi (Figure 1C). Parts of the testicular tissues from the same animals were also analyzed with IHC. ZIKV-ir, while negative for the testes isolated at 2 dpi, was positive in between the seminiferous tubules at 3 dpi and 4.5 dpi, possibly from infection of interstitial cells of the testis (Figure 1D–F). This clearly correlated with the presence of ZIKV RNA (Figure 1C) in the testes and indicated that ZIKV can establish infections in the testis as early as 3 dpi (but not 2 dpi). 

### 3.2. Prophylactic Treatment by SAB-155 of ZIKV Infection in STAT2 KO Hamsters

**Figure 2:**
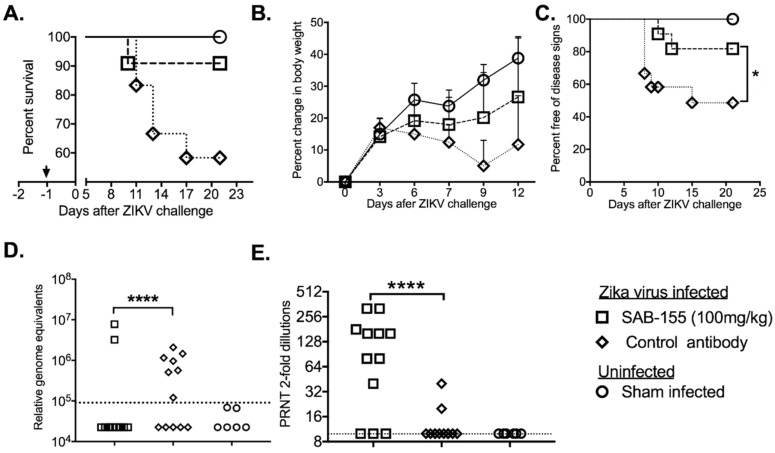
Prophylactic i.p. administration of SAB-155 to ZIKV infected *STAT2* KO hamsters. Equal numbers of male (*n =* 6) and female (*n =* 6) s.c. infected with PRVABC69 ZIKV. Sham infected controls had three each from both sexes. (**A**) Survival, arrowhead indicates treatment as one day before viral challenge (−1); (**B**) daily percent changes in the weights of surviving animals relative to the day of virus challenge; (**C**) percent free of eye disease, conjunctivitis; (**D**) Presence of ZIKV RNA in serum at 3 dpi. (E) Serum PRNT from serum of animals as in (**D**). *, *p* < 0.05; unpaired *t*-test; ****, *p* < 0.0001 by one-way ANOVA compared between SAB-155 vs. negative control antibody treatment. Dashed lines designate limit of detection in Figure 2D,E.

In order to evaluate the prophylactic efficacy of SAB-155 in protecting *STAT2* KO hamsters from ZIKV infection, equal numbers of age-matched male (*n =* 6) and female (*n =* 6) hamsters (6-weeks of age) were prophylactically treated by a single i.p injection of 100 mg/kg SAB-155 one day prior to ZIKV infection. Sham infected control groups with three hamsters from each sex were also included. As shown in Figure 2A, all of SAB-155-treated animals survived (except for one that died from eye bleeding procedures), whereas 42% (5 out of 12) animals in the control-antibody treated group succumbed. In addition, SAB-155-treated animals did not lose weight while the control-antibody treated animals started to lose weight at 3 dpi and continued to lose weight through 9 dpi (Figure 2B). *STAT2* KO hamsters exhibited conjunctivitis after ZIKV infection, which was significantly reduced in animals treated prophylactically with SAB-155: 50% (6 out of 12) of the control-antibody treated animals were free of conjunctivitis, while 81% (9 out of 11) of SAB-155 treated animals were free of eye disease (Figure 2C). Furthermore, 100% of the animals with conjunctivitis became moribund in the control antibody-treated group. Higher titers of ZIKV RNA were detected in the control antibody treated group than in the SAB-155 treated group (Figure 2D; PRNT data Figure 2E) suggesting prophylactic administration of SAB-155 at −1 dpi with a single dose of 100 mg/kg can inhibit ZIKV replication leading to effective prophylactic protection. 

### 3.3. Therapeutic Treatment by SAB-155 of ZIKV Infection in STAT2 KO Hamsters and Identifying the Effective Treatment Time Windows

**Figure 3:**
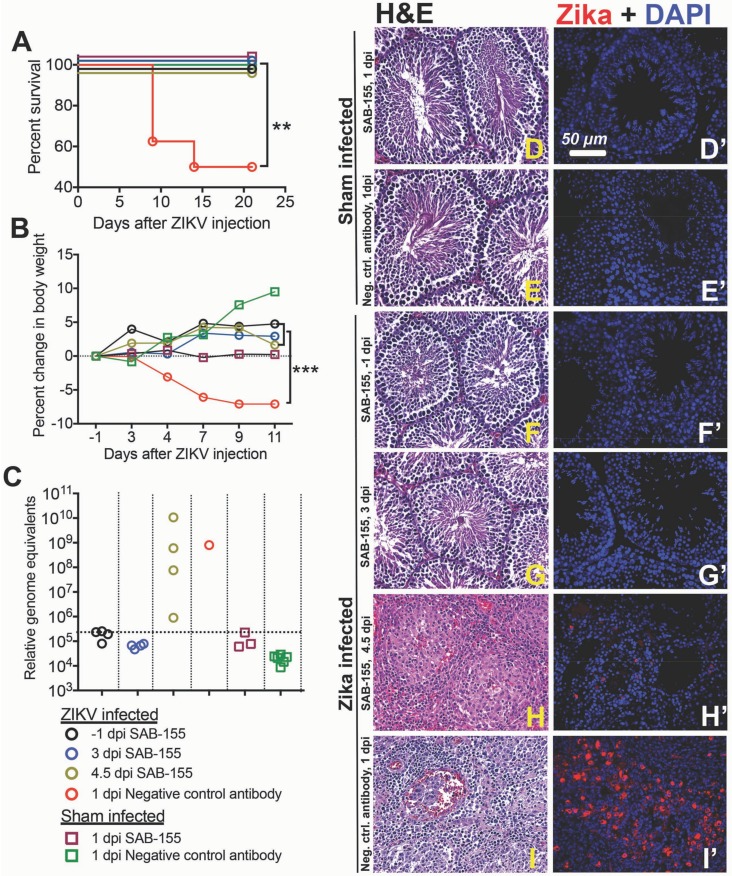
Identifying effective day of treatment with single dose of 100 mg/kg of SAB-155. Equal numbers of male (*n =* 4) and female (*n =* 4) *STAT2* KO hamsters infected with ZIKV PRVABC69 and treated i.p. with SAB-155 at different pre-and post-infection days either at −1, 3 or 4.5 dpi. Sham infected controls had 2 males and 2 female hamsters. (**A**) survival, (since five groups had 100% survival, data has been adjusted to display all groups); (**B**) temporal change in body weight relative to the day of virus challenge; (**C**) RT-PCR analysis of ZIKV RNA in testes of hamsters that survived up to at 21 dpi compared with sham infection; (**D**–**I**), Hematoxylin and eosin staining of cross section of testis tissue from ZIKV infected and SAB-155 treated *STAT2* KO hamsters. Arrows on the H pointing to the infiltrated cells. (**D’**–**I’**) immunohistochemical staining of ZIKV antigen (red color) in cross-section of testes tissue and DAPI (blue color) stained nuclei. **, *p* < 0.05; ***, *p* < 0.001; compared 1 dpi negative control antibody 100 mg/kg treated group with other groups, using one-way ANOVA test. Dashed lines indicate limit of detection in Figure 2C.

We subsequently conducted experiments to investigate the efficacy of therapeutic SAB-155 treatments on ZIKV infection and disease and to identify how late after infection an effective treatment can be administered. Equal numbers of age-matched (6-week) male (*n =* 4) and female (*n =* 4) *STAT2* KO hamsters were infected with ZIKV followed by treatment of SAB-155 via the i.p. route at either −1 dpi (treatment control group), 3 dpi, or 4.5 dpi. Sham infected control groups had two male and two female hamsters. As shown in Figure 3A, a single dose SAB-155 administered via i.p. injection at a dose of 100 mg/kg at −1, 3, and 4.5 dpi prevented *STAT2* KO hamsters from becoming moribund (Figure 3A), whereas the negative control antibody treated group started to become moribund at 8 dpi. Such data indicate that SAB-155 treatment as late as 4.5 dpi is effective in protecting hamsters from morbidity. It is interesting to note that, judged on a significant (*p* < 0.002) improvement in weight change, the therapeutic treatments at 3 and 4.5 dpi provided similar protection as prophylactic treatment (−1 dpi) (Figure 3B). It is unclear why the hamsters in the sham-infected group treated with SAB-155 on 1 dpi did not gain weight but we speculate that this was mostly caused by handling procedures during the experiments that have yet to be identified.

Next, in order to evaluate protective effect of SAB-155 against ZIKV infection in testicular tissues, RT-PCR and IHC were performed on the testes of surviving animals at the end of the experimental period on 21 dpi. As shown in Figure 3C, SAB-155 administered at −1 and 3 dpi to *STAT2* KO hamsters resulted in undetectable ZIKV RNA in their testes (Figure 3C). On the contrary, high levels of ZIKV RNA were detected in the testicular tissues collected on 21 dpi from the surviving male hamsters that were treated on 4.5 dpi (Figure 3C), even though animals treated at 4.5 dpi did not display mortality or body weight loss (Figure 3A,B). This suggests that ZIKV can cross blood-testis barrier and persistently infect seminiferous tubules and that SAB-155 treatment as late as 4.5 dpi is ineffective in viral clearance. The histology also showed that one of the four animals treated at 4.5 dpi had severe pyogranulomatous inflammatory infiltrates (Figure 3H and Table 1). ZIKV-ir indicates possible infection of interstitial cells, as well as cells lining the seminiferous tubules (Figure 3I’). The presence of ZIKV RNA (Figure 3C) in the testes corresponds with the histological lesions (Figure 3D–I) and ZIKV-immunoreactivity (Figure 3D’–I’). None of the *STAT2* KO hamsters treated with SAB-155 at −1 and 3 dpi exhibited any lesions (Figure 3F,G) nor showed ZIKV-immunoreactivity within the interstitium and seminiferous tubules (Figure 3F’,G’). Such data indicate that SAB-155 treatment as late as 3 dpi provided complete protection against ZIKV infection in the testis. Two out of four animals treated with SAB-155 on 4.5 dpi had pyogranulomatous inflammation in the interstitium and seminiferous tubules (Table 1) along with ZIKV positive cells (red color, Figure 3H’) even though the animal did not lose weight and survived to the end of the experimental period (Figure 3B). This suggests that *STAT2* KO hamsters can be used as a non-lethal animal model to study antivirals and vaccine treatment against ZIKV infection. 

### 3.4. Dose Titration of Therapeutic Treatment with SAB-155 against ZIKV Infection

**Figure 4:**
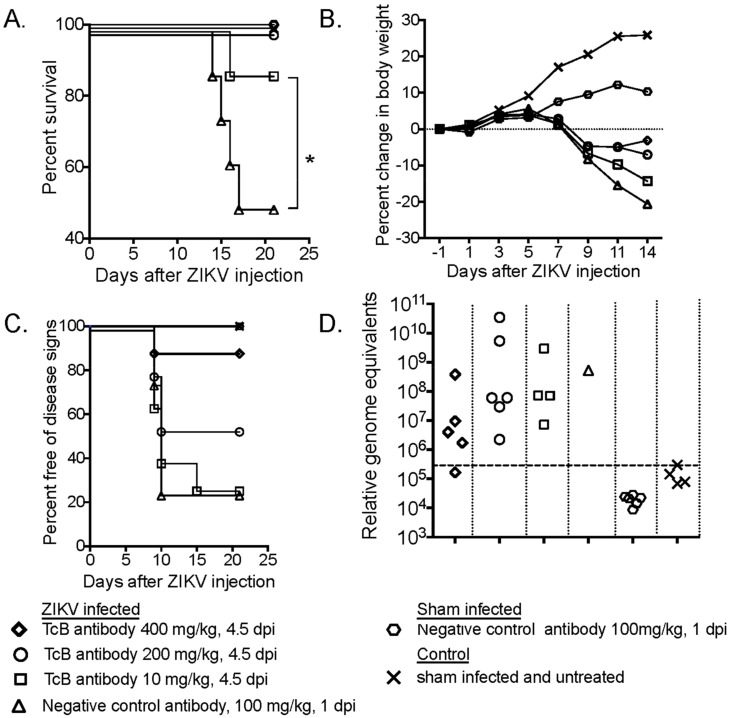
Identifying effective dose of SAB-155 treatment. Most of the groups had equal numbers of male (*n =* 4) and female (*n =* 4) *STAT2* KO hamsters s.c. infected with PRVABC69 and treated with different doses of SAB-155 at 4.5 dpi and negative control antibody at 1 dpi by i.p. administration. Groups treated with SAB-155 of 200 and 400 mg/kg had 6 male and 2 female hamsters. Sham infected controls had 3 each from both sexes. (**A**) Percent survival, (since three groups had 100% survival, Y axis was adjusted to display all groups); (**B**) percent change in body weight relative to the day of virus challenge; (**C**) percent free of conjunctivitis; (**D**) RT-PCR analysis of ZIKV RNA in testis at 21 dpi. *, *p* < 0.05 survival curve analysis (**A**). *P*-values obtained by comparing negative control antibody 100 mg/kg with 10 mg/kg of SAB-155 treated at 1 dpi. Dashed lines represent limit of detection in Figure 3D.

After demonstrating the efficacy of SAB-155 at a single dose of 100 mg/kg administered on 4.5 dpi at preventing morbidity, but not testicular infection, we investigated whether a single treatment with higher doses of SAB-155 could eliminate testicular infection and a lower dose of SAB-155 can still prevent animals from weight loss. Towards these goals, we tested 200 mg/kg and 400 mg/kg for the higher doses and 10 mg/kg for the lower dose. Survival (Figure 4A), weight loss (Figure 4B), percentage of free disease signs (Figure 4C), and RT-PCR on ZIKV RNA (Figure 4D) were evaluated for all treatment groups. We also performed H&E and IHC analyses on testicular tissues at 21 dpi for the higher dose groups (Table 1). A dose of 10 mg/kg SAB-155 treatment administered on 4.5 dpi prevented 88% (7 out of 8) animals from morbidity (Figure 4A). However, significant weight loss was observed in this low dose group of animals starting at 9 dpi (Figure 4B) and about 80% of animals showed disease signs (Figure 4C). In comparison, treatments with the higher doses of SAB-155 (200 mg/mL and 400 mg/kg) provided nearly 100% protection from morbidity (Figure 4A), while only marginally alleviating weight loss and disease signs (Figure 4B and 4C). As shown in Figure 4D, high levels of ZIKV RNA were still detected at 21 dpi in the testicular tissues of the hamsters treated at higher doses at 4.5 dpi, even though no mortality was observed. The detection of ZIKV RNA is consistent with the detection of pathological lesions and positive ZIKV-ir in this organ as summarized in Table 1. This indicates that at 4.5 dpi, neither of the two tested higher doses can provide complete protection against ZIKV infection in the testis. 

## 4. Discussion

Several mouse models of ZIKV infection, such as the stat2^-/-^ [18], A129 (*Ifnar*^-/-^), AG129, *Irf3*^-/-^*Irf5*^-/-^*Irf7*^-/-^ triple KO, and WT mice treated with type I IFN-blocking antibody have been reported with a variety of treatment strategies including small molecule antivirals and antibodies [19]. We recently reported that adult *STAT2* KO hamsters are highly susceptible to ZIKV infection and that ZIKV RNA titers were detected in the testis, kidney, brain and spinal cord of infected *STAT2* KO hamsters [10]. Compared to *stat2* KO mice which develop uniform lethal phenotype upon ZIKV infection, only about 50% of *STAT2* KO hamsters infected by ZIKV succumbed (Figure 2A), with the onset of death being delayed a few days in the hamster than in the mouse [18]. A side by side comparison between the *stat2* KO mouse model and the *STAT2* KO hamster model challenged with same strains of ZIKV would be interesting to investigate the difference between species in the development of the pathogenesis of ZIKV infection. Since *STAT2* KO hamsters often survive for the duration of the experimental period, i.e., 21 dpi, with viral RNA present in the testis, brain, and other organs, this unique hamster model provides an opportunity to test the efficacy of prophylactic and therapeutic treatments with anti-ZIKV antibodies. 

Currently, there are no FDA approved ZIKV vaccines or antiviral drugs available, therefore, there is an urgent need for the development of efficient control strategies for this emerging pathogen. A number of antivirals have been shown to be active against ZIKV infection in immunodeficient AG129 [12,20], A129 (*Ifnar*^-/-^) [21], and immunocompetent C57BL/6 mouse models [22]. Apart from the antivirals, antibodies with inactivated Fc receptors have also been shown to be protective in both immunocompromised and immunocompetent pregnant rodent models. Convalescent sera from ZIKV-infected patients [23], monoclonal antibody from DENV patients [24], and ZIKV-117 mAb from human antisera [25] were tested against different strains of ZIKV virus in several mouse models. Hundreds of monoclonal antibodies targeting the E protein of ZIKV with neutralization activity of <1µg/mL, either derived from human patients or mice, have also been identified [26]. In primate models, the administration of a cocktail of potent neutralizing monoclonal antibodies collected from ZIKV-infected patients completely prevented serum viremia [27]. In the present study, we used a unique hamster model to investigate the prophylactic and therapeutic effects of SAB-155 against ZIKV infection. We were particularly focused on the potential of SAB-155 in eliminating ZIKV in the testis, and to gather some preliminary scientific information on the potential of using SAB-155 to treat sexual transmission. 

We first characterized the kinetics of viremia, the development of neutralizing antibodies, and the presence of virus in *STAT2 KO* hamsters following ZIKV infection. We established that endogenous anti-ZIKV neutralizing antibody responses are elicited by 3 and 4 dpi. Viral RNA and ZIKV-ir in the testis are also detectable during this time window. We then tested whether a single dose of SAB-155 administered prophylactically at −1 dpi would be able to protect the hamsters from morbidity, weight loss, and conjunctivitis. Our results showed that prophylactic treatment at −1 dpi at a dose of 100mg/kg of SAB-155 was effective in preventing hamster mortality (Figure 2A) and weight loss (Figure 2B), as well as a reduction in disease signs (conjunctivitis; Figure 2C). Based on these data, it appears that prophylactic treatment with TcB antibodies can be an effective countermeasure against ZIKV infection. However, further studies in non-human primate models or in clinical trials will need to be conducted to test this possibility.

Our study on the kinetics of viremia and the time frame to effectively treat ZIKV infection with SAB-155 also provided valuable data on when therapeutic intervention could be effective. It is striking that treatment with a single dose of 100 mg/kg SAB-155 as late as at 4.5 dpi can still fully protect animals from morbidity, even though these animals had high levels of ZIKV RNA and positive ZIKV-ir in their testicular tissues on 21 dpi. Furthermore, our data showed that a single therapeutic treatment with SAB-155 at or before 3 dpi prevented animals from being ZIKV-ir in the testis and developing histopathological lesions provided the possibility that sexual transmission of ZIKV could be prevented if the treatments can be administered early enough post infection. 

In summary, we first demonstrated ZIKV infection and immunotherapy in a new rodent animal model, the *STAT2* KO hamsters. According to the FDA animal efficacy rule, *the development of therapeutic interventions against serious conditions, such as those caused by ZIKV, should be demonstrated to be efficacious in more than one animal species*. Second, we demonstrated that SAB-155 is highly effective, both prophylactically and therapeutically, in treating ZIKV infection in this hamster model. In contrast to other animal-derived monoclonal antibodies, SAB-155 is fully-human (non-immunogenic to patients), polyclonal (recognizing multiple antigens on a virus to effectively reduce or eliminate viral evasion caused by viral mutations) and can be produced very quickly and in large quantities (up to 600 g fully human immunoglobulin can be produced per Tc bovine per month; our unpublished observations). These advantages over other antiviral therapies could potentially make SAB-155, as well as the TcB platform, a powerful countermeasure in fighting ZIKV infection. It is worth noting that TcB derived fully human polyclonal antibodies elicited against several other viruses or bacteria have been shown to be effective to treat infections caused by various infectious pathogens [5,6,11,28,29,30,31]. Some of these TcB antibodies have already been proven to be safe and efficacious in human clinical trials [31,32]. The anti-ZIKV TcB antibodies reported in this study are yet to be tested in rigorous human clinical trials to be proven effective in treating ZIKV in human infections.

## Figures and Tables

**Table 1:** Effect of SAB-155 antibody treatment on testis of Zika virus infected *STAT2* KO hamsters.

		Treatment Day	SAB-155 Antibody Dosage	Testis			
				ZIKV RNA by RT-PCR	Morphological Diagnoses *	Immunohistochemistry	Lesions/Total No. of Male Hamster Necropsied
Identifying effective day of antibody treatment **	SAB -155 treatment	−1 dpi	100 mg/kg	Below the limit of detection (<10^5.5^)	No significant lesions	4/4 No ZIKV infected cells found	0/4
		3 dpi	100 mg/kg	Below the limit of detection (<10^5.5^)	No significant lesions	4/4 No ZIKV infected cells found	0/4
		4.5 dpi	100 mg/kg	10^6^–10^10^	Pyogranulomatous inflammatory exudate infiltrates.	1/4 animal had ZIKV antigen in ST.	1/4
	Negativecontrols	1 dpi	Irrelevant Ab. 100 mg/kg	10^9^	Diffusely pyogranulomatous inflammatory exudate infiltrates expand and replaces the interstitium of ST.	2/4 animal had ZIKV antigen in ST. 2/4 animal died before 21 dpi.	2/2
Uninfected controls **		1 dpi	100 mg/kg	Below the limit of detection (<10^5.5^)	No significant lesions	No ZIKV infected cells found	0/4
		1 dpi	Irrelevant Ab. 100 mg/kg	Below the limit of detection (<10^5.5^)	No significant lesions	No ZIKV infected cells found	0/4
Identifying effective dose of antibody treatment ***	SAB-155treatment	4.5 dpi	200 mg/kg	10^6^–10^11^	1/6 with a minimal pyogranulomatous orchitis.4/6 with a severe pyogranulomatous orchitis	6/6 animals had ZIKV antigen in ST.	6/6
		4.5 dpi	400 mg/kg	10^5^–10^8^	1/6 with a severe pyogranulomatous orchitis.	6/6 animals had ZIKV antigen in ST.	6/6
	Negative controls	4.5 dpi	Irrelevant Ab. 400 mg/kg	10^6^–10^10^	5/6 with a severe pyogranulomatous orchitis.	5/6 animals had ZIKV antigen in ST.1/6 animal had coagulative necrotic testis. 1/6 animal died before 21 dpi.	5/6
		1 dpi	Irrelevant Ab. 100 mg/kg	10^8^	3/4 animal died before necropsy. One animal survived with severe pyogranulomatous orchitis.	3/4 animal died before necropsy.1/4 animal with heavy ZIKV+ve ST.	1/1
UninfectedControls ***		1 dpi	100 mg/kg	Below the limit of detection (<10^5.5^)	No significant lesions	No ZIKV infected cells found	0/6
		1 dpi	Irrelevant Ab. 400 mg/kg	Below the limit of detection (<10^5.5^)	No significant lesions	No ZIKV infected cells found	0/6
		1 dpi	Vehicle	Below the limit of detection (<10^5.5^)	No significant lesions	No ZIKV infected cells found	0/6

*: Hematoxylin and eosin stained slides read by board certified veterinary pathologist. **: One each testis processed for RT-PCR and IHC. ***: same testis tissue processed for RT-PCR and IHC.
